# Estimating damage costs of flooding on small- and medium-sized enterprises in Kigali, Rwanda

**DOI:** 10.4102/jamba.v11i1.755

**Published:** 2019-09-18

**Authors:** Aime Tsinda, Christian Kind, Janto S. Hess, Roger Mugisha, Alfred R. Bizoza

**Affiliations:** 1 College of Education, University of Rwanda, Kigali, Rwanda; 2 Adelphi, Berlin, Germany; 3 Institute of Policy Analysis and Research, Kigali, Rwanda; 4 College of Agriculture, Animal Sciences and Veterinary Medicine, University of Rwanda, Kigali, Rwanda

**Keywords:** flooding, enterprises, climate change, damage costs, Kigali

## Abstract

In the past, Kigali has frequently experienced heavy rain events. These have often led to flooding, which also affected businesses. In the face of climate change, such events can become more frequent and can threaten economic development. To determine if more action is needed to protect businesses from flooding, we assessed how many businesses have suffered damages from floods in certain years in a certain area of Kigali. We also gathered information on how businesses were affected, how they are preparing for flooding and what support they are seeking. We developed and piloted a survey, a standardised questionnaire for gathering information on the relevance of flooding for businesses. The survey was then conducted among 350 businesses in Kigali asking business owners about their experiences with flooding in recent years. Eighty-one per cent of businesses have been affected by floods in 2013 and 2014. The annual damage costs resemble 22% of the total net profit of the businesses in the area. The most common damages were damages to goods that were to be sold and damages to buildings. The extent of past flood damages warrants action on flood risk management, both by businesses and citizens, as well as by city officials. Suitable actions range from increasing awareness about suitable protection measures to upgrading the sewage system.

## Introduction

Worldwide, extreme precipitation events, which can lead to landslides and floods, are one of the most severe hazards to human settlements and have caused considerable economic and social costs. In the light of progressing climate change, the likelihood of such events will further increase (IPCC 2014; Stocker 2014). In combination with continuous population growth, urbanisation trends and insufficient consideration of extreme weather hazards in development planning processes, it is expected that the magnitude of damages and the resulting costs will rise accordingly (Field et al. 2012). This will, in particular, be the case in countries with developing economies and hilly topographies, such as Rwanda (IPCC 2014; World Bank Group 2016). Located in sub-Saharan Africa, Rwanda is the most densely populated nation in the region (GIZ 2014). Limited reliable historical weather data or precipitation projections are available for Rwanda. The existing sources reveal that there was no significant trend of changing precipitation patterns between 1931 and 1990 (MINIRENA 2011). However, the frequency of heavy rainfall events has increased and is expected to further intensify because of climate change and climate variability, such as the El-Nino Southern Oscillation (FCFA 2014; World Bank Group 2016). Overall, it is estimated that the rainy seasons (from September to November and March to May) become shorter, while precipitation is intensifying and extreme events such as floods are likely to increase in frequency (SEI 2009).

The economy of Rwanda is vulnerable to the impacts of climate changes whose additional net economic costs are estimated to be almost 1% of gross domestic product (GDP) each year by 2030 (SEI 2009). So far, floods and landslides after heavy rain have been the natural disasters causing the most damages (FCFA 2014). Considering that around 98% of Rwanda’s companies can be classified as small- and medium-sized enterprises (SMEs, including micro), it is important for economic development to understand businesses’ specific vulnerabilities and the damages they face as a result of extreme weather events (GIZ 2014). This is of particular relevance with respect to flooding: when analysing historical data and projected economic implications of climate change for Rwanda, it can be said that flood events are the main source of costs from hazards for their economy (SEI 2009).

Thus, analyses of the extent of flood damages for businesses are of importance as they can provide policy makers and entrepreneurs with information which can be used as a basis for reducing economic loss from flood damages, for example, via improved flood risk management in affected locations (Merz et al. 2010). However, estimating the full range of economic costs from natural disasters is difficult, both conceptually and practically, particularly in locations with limited available baseline data (Kousky 2012; Merz et al. 2010). Local circumstances need to be taken into account in developing adequate methodologies. Although flood damage assessments can direct disaster management and climate change adaptation activities in a valuable way, it has not received much scientific attention in the context of Rwanda. While flooding and flood protection in Kigali are receiving more attention from scientists in recent years (Habonimana et al. 2015; Mugisha 2015; Munyaneza, Nzeyimana & Wali 2013), a web-based analysis of existing peer-reviewed literature leads to the conclusion that there are no scientific publications that touch on the actual costs of flood damages for businesses in Rwanda. Most publications focus on possible levels of future floods or on technical fixes without looking into the extent of damages caused by flooding.

Against this background, the article aims to quantify business-related damage costs incurred from flooding and the determinants of impacts on businesses in the catchment area of the Nyabugogo River in Kigali in recent years. These investigations can benefit science and policy-making in different ways: they can add to methodology development for assessing damages costs for businesses, provide policy makers with a baseline of damage costs against which flood risk management options can be selected and inform entrepreneurs about the most effective measures for dealing with floods in the area.

### Small- and medium-sized enterprises, vulnerability and resilience

In a developing country context, SMEs can be credited with the potential to increase economic growth, alleviate poverty, foster development and decrease income inequalities (USAID 2008). These positive attributes are oftentimes related to the small size, innovative potential and employment (or self-employment) opportunities, even for people with low levels of education. However, SMEs are also more vulnerable to natural hazards than larger firms because of constraints of human and financial resources to recover after disastrous events as well as limited access to loans or government training or programmes (Ballesteros & Domingo 2015; Han & Nigg 2011; UNDP 2013). The latter particular counts for the informal sector within a country. On the contrary, the inherent flexibility of SMEs, because of their size and comparably low operational costs, shape their resilience (Ballesteros & Domingo 2015; Han & Nigg 2011; UNDP 2013).

There are a range of characteristics of SMEs that point to higher vulnerability^1^ of such companies towards natural hazards in comparison to larger firms, which include (UNDP 2013; UNESCAP 2013): (1) limited financial capital, (2) small number of employees, who are potentially unavailable after disasters, (3) limited mobility of production sites, (4) limited access to loans and (5) unaffordable or unavailable insurance cover. Furthermore, the vulnerability of SMEs in developing countries can further get exacerbated through (ILO 2002; Perry et al. 2007; UNDP 2013): (1) informality that limits their access to government-led support programmes, (2) a lack of compliance with norms and regulations that can increase disaster risks and (3) as young workers and women are over-represented in informal enterprises, they can be considered more vulnerable when compared to other groups (ILO 2002; Perry et al. 2007).

However, it can also be claimed that the characteristics of SMEs, like their flexibility and limited capital needs for operation and recovery, entail an inherent resilience (Ballesteros & Domingo 2015; UNDP 2013). Resilience in this context can be understood as (UNISDR 2009):

[*T*]he ability of a system, community or society exposed to hazards to resist, absorb, accommodate to and recover from the effects of a hazard in a timely and efficient manner, including through the preservation and restoration of its essential basic structures and functions. (p. 24)

Other concepts go beyond this bounce-back concept and look at what capacities are needed for businesses to return stronger than before. Most reviewed literature though indicates that this inherent resilience does not outweigh the vulnerability factors of SMEs in a developing country context.

Therefore, there is a need for SMEs to prepare for disasters through identifying their vulnerabilities and enhancing their resilience. This process ‘requires partnerships and cooperation among the firms, public and other private organizations’ (Ballesteros & Domingo 2015:8). The government can play a pivotal role in creating a policy and support framework that enables SMEs to effectively enhance their resilience, for example, through early warning systems, training programmes or incentivising positive behaviour (Ballesteros & Domingo 2015).

In the aftermath of a flood event, it is important to minimise the recovery time of SMEs to avoid further marginalisation of affected people and sustain income opportunities. Asgary, Anjum and Azimi (2012) surveyed 500 small businesses in three flood-affected provinces in Pakistan and found that the ‘provision of minimum government and non-governmental support can enhance the speed, quality and sustainability of the small businesses disaster recovery’ (Asgary et al. 2012:46). After the major flooding in Thailand in 2011, the government provided a subsidy for employees and workplaces to maintain income, short-time training courses on business skills with food allowances, as well as a soft loan programme (UNESCAP 2013). These measures in conjunction with support from non-governmental actors and the workforce itself helped to maintain jobs and minimise recovery times of affected SMEs. These two examples indicate the importance of cooperation and collaboration between stakeholders to enhance the resilience of SMEs.

### Estimating costs of floods

Extreme weather events, such as heavy rain events triggering floods, can lead to a variety of damages and resulting costs (Table 1). Costs from extreme weather events can be categorised as being costs from direct and indirect damages. Furthermore, these cost types include tangible and intangible costs (Kousky 2012; Messner et al. 2007; Meyer et al. 2013).

**TABLE 1:** Typology of damage costs.

Variable	Tangible	Intangible
Direct damage	Physical damage to assets such as buildings, infrastructure, machinery or inventories	Loss of life, health effects or loss of ecosystem services
Indirect damage	Disruption of production processes at supply firms or traffic disruptions	Inconvenience of post-flood recovery, reputational damages and increased vulnerability to future hazards

*Source*: Adapted from Kousky, C., 2012, *Informing climate adaptation: A review of the economic costs of natural disasters, their determinants, and risk reduction options. (No. RFF DP 12–28)*, Discussion Paper, Resources for the Future, Washington, DC; Messner, F., Penning-Roswell, E., Green, C., Meyer, V., Tunstall, S. & Van der Veen, A., 2007, *Evaluating flood damages: Guidance and recommendations on principles and methods*, FLOOD site-Report T09-06-01, Wallingford, UK; Meyer, V., Becker, N., Markantonis, V., Schwarze, R., Van den Bergh, J.C.J.M., Bouwer, L.M. et al., 2013. ‘Review article: Assessing the costs of natural hazards – State of the art and knowledge gaps’, *Natural Hazards and Earth System Sciences* 13, 1351–1373. https://doi.org/10.5194/nhess-13-1351-2013; Kuik, O.J., Bucher, B., Catenacci, M., Karakaya, E. & Tol, R.S.J., 2006, ‘Methodological aspects of recent climate change damage cost studies’, *Integrated Environmental Assessment and Management* 8(1), 19–40.

The most immediate physical effects of extreme events on assets, human beings or the environment are characterised as direct costs. The results of damages to buildings, infrastructure, machinery or inventories are among the most visible impacts of extreme weather events. Indirect damage costs are costs that arise from reductions in demand if customers are directly affected by an extreme weather event or from the interruption of production processes because of a lack of production inputs provided by supply firms that are either directly affected by an extreme weather event or that cannot supply production inputs to the business under consideration because of traffic disruptions caused by damages to transport infrastructure.

Direct damage costs are often measured using susceptibility functions (Meyer et al. 2013), base data on insured losses associated with physical flood damage (Patankar & Patwardhan 2016) or utilise remote sensing techniques and geographical information systems (Haq et al. 2012; Su et al. 2005). Indirect costs can be measured using economic models or firm and household-level surveys relating to past events (Meyer et al. 2013). Micro level surveys also offer an alternative to measure direct damage costs in places where insurance coverage is low and the informal sector extensive (Patankar & Patwardhan 2016) – as is the case in Rwanda.

Overall, assessments of direct and indirect costs of damages from extreme weather events can be conducted on different geographical as well as temporal scales. The methodological approach used for cost evaluation is determined by the selected spatial level. While macro approaches of damage evaluation on a national or even international level (Bizimana et al. 2009; Defra 2001; Feyen & Watkiss 2011; World Bank Group 2012) require a comparably low amount of input data and resources per unit of area, estimations on a meso level (BWK 2001; Defra 2009; DWA 2008; Mai et al. 2004; Ministrie van Verkeer en Waterstaat 2005; State of California 2012) or micro (local) level (DWA 2008; Hallegatte 2009; Hammond et al. 2015; Malte 2013; Vilier, Kok & Nicolai 2014) require detailed data. The same applies to demands for precision of the underlying information as the level of abstraction decreases (Messner & Meyer 2005). These diverging characteristics are mainly determined by the objectives of damage evaluation. Evaluation on a national or regional level is mainly used to justify the allocation of public funding, and therefore approximate evaluations with comparably low precision that allows a comparison of different regions are sufficient. If the objective is to inform households or firms, the evaluation should be precise as wrong estimates can lead to an underestimation of risks and to insufficient preventive action (Messner et al. 2007), whereas data availability and country-specific circumstances need to be taken into account.

Data to be collected for the determination of the value of assets at risk at a micro level require a categorisation of the building type and usage. This can further be divided by subcategories such as the business sectors, age of the building, inventory of machinery and other equipment, type of materials or goods, area and use of the ground floor and/or basement, or elevation based on threshold for floodwater intrusion, as well as height above ground level (Messner et al. 2007; Thieken et al. 2008). Past flood events can also provide valuable information on the potential costs from flooding. In addition to the collection of data on the assets at risk, characteristics of the past flood event, information on the actual damage and about damage reduction measures that existed at the time of the event have to be collected when evaluating past events. Flood characteristics encompass the date, duration and area of the event, the flood type (e.g. riverine flood or flash flood), the maximum water level, as well as the possible type and grade of contamination of floodwater.

Unlike direct damage costs, the assessment of business disruption costs is subject to a less straightforward approach. Business disruption costs have to be assessed by either using sector-specific reference values, by comparing production outputs for years when the business was affected by an extreme weather event with a year without such an event or by calculating production losses using a fixed share of direct damages (Mechler 2009; Meyer et al. 2013). For the assessment of indirect costs, a variety of methodological approaches are available encompassing surveys, econometric modelling, input–output modelling or computable general equilibrium (CGE) modelling. Among other methods, surveys can provide information on the structure of a businesses’ supply chain and critical elements within that supply chain as well as estimates or experience-based cost assessments of supply chain disruptions (Meyer et al. 2013). A survey-based investigation can further allow receiving information about potential root causes of business interruptions, vulnerabilities and resilience factors of investigated entities (e.g. businesses, households or infrastructure).

In conclusion, it can be said that there is a wide range of theoretical approaches and tested methodologies for estimating damage costs of extreme weather events, such as floods triggered by heavy rains. However, it is apparent that specific circumstances, objectives, spatial scales and time frames shape the selection process of methodologies. Data availability is among the key determinants for selecting a suitable methodology.

## Methodology

Against this theoretical background and the reviewed empirical assessments, it is apparent that conducting a survey on a single unit of analysis (businesses) is an adequate methodology for the purposes outlined above that allows gathering data on damage cost estimates and insights into entrepreneurs’ behaviour in the Rwandan context. There are three limitations of this approach: (1) Few business owners possess written records of flood damages and thus had to rely on their memory and estimations of costs to reply to the questions. (2) Business owners do not have much time in their daily work to respond to questionnaires; thus, some answers might have been rushed. (3) The exact locations of the responding businesses were not recorded as this would have made it impossible to ensure anonymity to the respondents. Anonymity, however, was important because sensitive information like revenues were gathered. To address the first two limitations, the survey only focuses on damages from the years 2013 and 2014.

**FIGURE 1:**
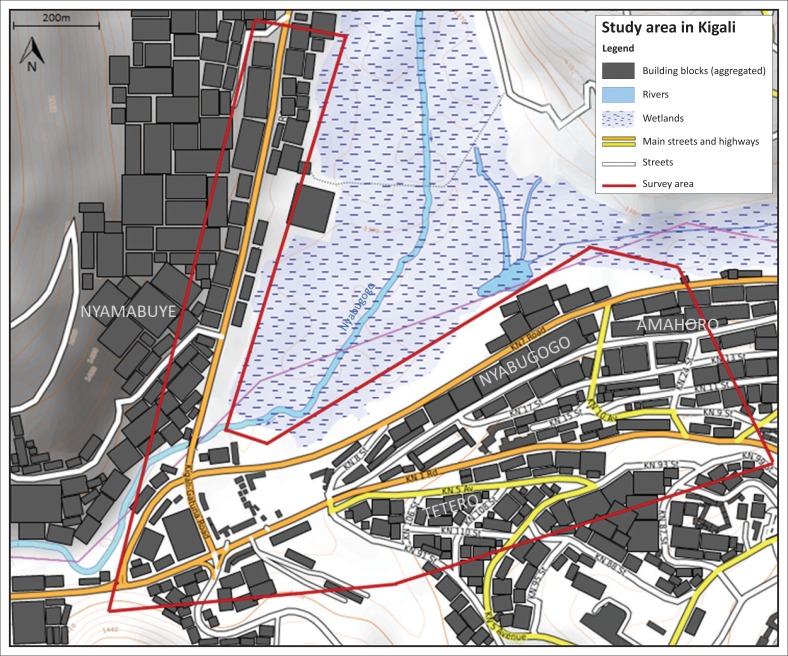
Map of the surveyed area. Map credits: ^©^OpenStreetMap-contributors (SRTM); Map design: ^©^OpenTopoMap (CC-BY-SA), changes made: Buildings added, individuals buildings are aggregated, area encircled in red shows surveyed area.

The field research was carried out in the area around the Nyabugogo River within the catchment of Nyabugogo–Gatsata–Kimisagara–Giti Kinyoni, City of Kigali, Rwanda (Figure 1). This area was selected because it has a high density of SMEs and has frequently been struck by extreme weather events in recent years. Local experts estimate that there are around 1000 businesses in the target area.

The data collection was undertaken in a two-stage process: (1) transect walks around the study area to observe enterprise styles and construction features, such as used materials, as well as geographical features such as slopes and swamps (propensity to flooding for example); and (2) a quantitative survey, for which structured interviews with SMEs based on a standardised questionnaire were conducted. The structured interviews for the survey were conducted between 19 August 2015 and 07 September 2015.

A range of studies successfully applied comparable firm or household-level surveys to estimate flood damage costs and impacts (Khandlhela & May 2006; Patankar & Patwardhan 2016), in perspective of loss and damage from climate change (Warner & Van der Geest 2013), and related to sea-level rise, saltwater intrusion and coastal erosion (Monnereau & Abraham 2013; Rabbani, Rahman & Mainuddin 2013). Most of these studies are based on a sample size of around 360 cases per target region.

For the estimated number of SMEs in the Kigali river catchment, the sample size needed, according to Slovin’s formula, should include at least 286 businesses to allow drawing representative conclusions (with a confidence level of 95%) for all businesses in the area (Guilford & Fruchter 1973). To increase the validity and avoid the risk of having an unrepresentative sample, for example, through wrong estimates or new SMEs settling in the area, 360 businesses were targeted. Out of these 360 businesses, 353 businesses (98%) were interviewed giving a non-response rate of only 2%.

All of the businesses in the sample have less than 100 workers, and thus can be classified as being SMEs (Ayyagari, Demirguc-Kunt & Maksimovic 2011). The selection of interviewed SMEs was based on systematic random sampling, during which the interviewers approached every third business on both sides of the street (Bryman 2012).

### Structured interviews

The methods of survey research in the form of a structured interview of individuals were applied during the investigation as the method is versatile and efficient (Schutt 2012). Despite these positive attributes, surveys are prone to inaccuracies, for example, through (Schutt 2012): (1) measurement error, which describes the effort it takes to answer a question; (2) non-response, which can be minimised by choosing the right survey method; and (3) inadequate samples, which includes the sample size as well as the characteristics of the interviewees. All of these problems were considered in the preparation of the data collection, for example, by using Slovin’s formula to calculate the right sample size and conducting a pre-test of the questionnaire.

Furthermore, the questionnaire was carefully designed to avoid inaccuracies and measurement errors, keeping in mind that questions should be clearly phrased and neutral (Gray 1999). Correct phrasing and a prudent selection of questions can minimise the risk of bias (Bryman 2012; Schutt 2012). Most of the questions were closed questions with a set of predefined, short answer possibilities, whereas multiple answers were allowed where suited (Bryman 2012).

When designing the interview schedule, refining and testing the questions included is a crucial step (Schutt 2012). This should be performed through a pre-test, which can take on different forms (Dillman 2000). Options include discussing the survey with other experts, such as researchers and individuals or key figures in the field of interest or testing the draft questionnaire with individuals who belong to the target group (Schutt 2012). Hence, the survey questionnaire was both discussed with experts in the field as well as tested with business owners just outside of the targeted zone. Afterwards, the feedback from both groups was used to optimise the questionnaire.

The questionnaire was then administered face to face by three trained interviewers in the preferred language of the respondents, who were asked to respond on behalf of the owner of the enterprises. Three call-backs were made before an enterprise was recorded as a non-response.

### The approach used to estimate damage costs

**FIGURE 2:**
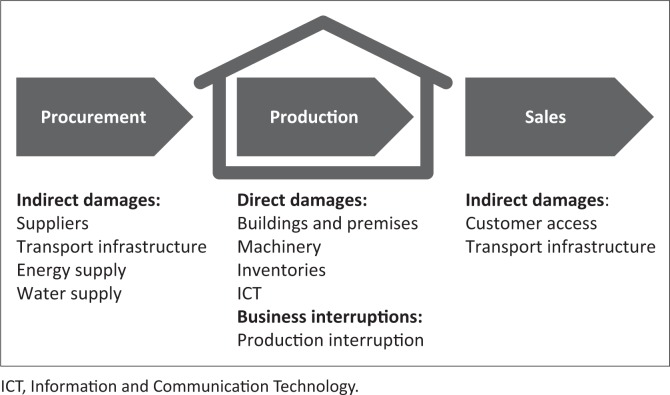
Damage types and assets at risk included in the survey along the value chain.

The damage cost estimation in this study focuses on a micro (local) level. The questionnaire was designed to gather baseline information on the socio-economic characteristics of the sampled enterprises, flood-related damages (both direct and indirect damages, and business interruptions) in 2013 and 2014 as well as information on flood protection measures the enterprises implemented, or not, to enhance their resilience against flooding. This information enabled an investigation of damage types on all three broadly defined stages of a value chain, namely procurement, production and sales (Figure 2). While it would have been interesting to cover a larger period of time, but going further back would have entailed a high risk of memory problems distorting the results.

The damage costs themselves are separated into direct and indirect damage costs. As it was expected that the interviewees would not be able to precisely reflect estimates on all costs for their enterprise triggered by floods in recent years, questions about damage costs always referred to the most severe flooding event in the two years between 2013 and 2014 that the respondents could recall. This method of self-reported costs enabled a rough estimate of the costs of high-impact events on businesses in the area for each year. The estimates of indirect damage costs are based on the number of days of business interruptions after the strongest (most severe) flood event, in 2013 and 2014, and the estimates by the interviewees of revenue loss per day. Further investigation of the data set, utilising the analytical software SPSS, was undertaken in order to receive insights into the relation of business type and size, damage experienced and disaster risk reduction measures implemented or interested in.

### Ethical considerations

This article followed all ethical standards for research without direct contact with human or animal subjects.

## Results and discussion

### Socio-economic characteristics of sampled businesses

The following section provides insights on the overall socio-economic characteristics of the sample of 353 SMEs surveyed in Nyabugogo, Kigali, Rwanda. In the sample, among the most dominant types of enterprises were car item retailers with 40.2% (142 SMEs), which were followed by retailers of daily use items with 35.1% (124). The other types of enterprises were far less in number. These were wholesalers of food (7.6%, 27 SMEs), manufacturers (6.8%, 24 SMEs), hotels and restaurants (4.2%, 15 SMEs), service providers (3.4%, 12 SMEs), and hardware stores (2.5%, 9 SMEs). This distribution reflects the general image of the area as a main marketplace, particularly for car parts, in that part of Kigali.

Among these businesses, only 2.5% (9) own the premises of the enterprise. The other 97.5% are renting the premises. Ownership of property of the enterprise can be a determining factor in the motivation of entrepreneurs to engage with disaster risk reduction measures. Compared with renters or leasers, property owners may feel that their assets are at higher risk, and thus they may be more concerned about vulnerability and disaster reduction (Han & Nigg 2011).

The size of the enterprises (in terms of number of employees) ranged from one employee to more than six employees, with the majority of enterprises having two employees (47%, 166 SMEs, including the owner), followed by one employee (31.4%, 111 SMEs), three to five employees (16.1%, 57 SMEs) and more than six employees (5.4%, 19 SMEs). Thus, the vast majority of enterprises surveyed (78.4%) operate with no more than two workers and could be classified as being small, or even micro enterprises.

All surveyed SMEs were asked to reveal their net profits. More than half of the sample (51.3%) earned less than 100 000.00 RWF (Rwandan franc) (about $140.00) per month. Another 41.4% earned a monthly profit between 100 000.00 RWF and 500 000.00 RWF. Only seven enterprises earned more than 500 000.00 RWF (around $700.00). Based on these self-declared net profits, the overall monthly profit for all businesses surveyed is estimated to be 53 425 000.00 RWF (around $75 000.00). The combined *annual* profit of the SMEs in the sample is 641 100 000.00 FRW (around $890 000.00).^2^

### Cost estimates of direct and indirect damage costs for enterprises through flooding

The entrepreneurs in the Nyabugogo catchment area recognise flooding as the main hazard for their businesses: when asked which of the four disasters they consider most dangerous for their operations, 88.7% mentioned flooding, followed by fire in the building (9.3%), windstorms (1.1%) and landslides (0.8%). The recognition of this hazard seems to be in line with the experience of businesses regarding flooding in the area. Eighty-one per cent (284 businesses) were affected by flooding at least once between 2013 and September 2015. In the period between 2013 and 2014, the surveyed enterprises were most often affected (i.e. suffered any damage) by flooding in the year 2013 (Table 2); with 181 SMEs indicating that they have been affected one to two times and 25 businesses three to four times in that year. These two years were selected because they were the most recent years at the time of the survey in 2015. This had the advantage that respondents could still remember events and damages relatively well.

**TABLE 2:** Frequency of enterprises being affected by flooding between 2013 and 2014.

Frequency	2013	2014
Zero times	147	192
One to two times	181	154
Three to four times	25	7

**Total**	**353**	**353**

These findings correlate with the answers given by the interviewees about the year in which they experienced the most severe flood impact (Table 3). A total of 53.5% (189 SMEs) of the sampled enterprises experienced the severest flood in 2013. Among all the businesses surveyed, only 69 did not experience any flood damage in the years 2013 and 2014.

**TABLE 3:** Year of the most severe flood impact on the enterprises between 2013 and 2014.

Year	Frequency	Percentage
2013	189	53.5
2014	95	26.9
Total	284	80.5
Missing	69	19.5

**Total**	**353**	**100**

Based on these most severe flood events experienced by 284 businesses of the sample, the direct and indirect damage costs for the enterprises were calculated (see below). Among the most common direct flood damages were damages to items that were supposed to be sold (experienced by 74% of affected businesses, multiple answers possible), damage to building or premises (e.g. the door or walls of the building were damaged; 36.8%), damage to equipment or machinery (e.g. cash register or tools; experienced by 18.8% of affected businesses) and damage to production inputs (e.g. raw materials like wood; 3.1%).

The estimated damage costs of these single most severe flood events in 2013 and 2014 for businesses in the area amount to 144 800 000.00 RWF (around $200 000.00) (Table 4). Most of these damage costs (122 700 000.00 RWF; around $170 000.00) occurred in 2013. The flood damage was significantly less in 2014, despite the fact that about half the amount of SMEs declared their most severe event in that year compared to 2013.

**TABLE 4:** Estimation of direct (physical) damage costs based on the most severe event for each enterprise by year cross-analysed with estimated direct damage costs for those events (all amounts in Rwandan franc).

Direct damage in RWF	2013	Costs 2013†	2014	Costs 2014†	Total cost†
< 100 000.00	64	3 200 000.00	57	2 850 000.00	6 050 000.00
100 001.00–500 000.00	48	12 000 000.00	13	3 250 000.00	15 250 000.00
500 001.00–1 000 000.00	20	15 000 000.00	8	6 000 000.00	21 000 000.00
1 000 001.00–5 000 000.00	34	85 000 000.00	4	10 000 000.00	95 000 000.00
5 000 001.00–10 000 000.00	1	7 500 000.00	0	-	7 500 000.00
Not declared	22	-	13	-	-

**Total**	**189**	**122 700 000.00**	**95**	**22 100 000.00**	**144 800 000.00**

RWF, Rwandan franc.

† , Estimated damage costs are based on the middle amount within each category (e.g. 750 000 for the category 500 001–1 000 000) and their related frequencies per year, whereas for the lowest category, an amount of 50 000 was used.

Additional to these tangible direct damages, anecdotal evidence shows that some people were injured during the flood events in the area. Furthermore, the floods negatively affected ecosystem services, such as the provision of local raw materials like wood.

Businesses were asked to estimate the profit losses through business closures after the flooding event that was most severe to them between 2013 and 2014 (Table 5). The most common reasons for business interruptions were that neither customers nor employees could access the premises anymore (mentioned by 216 businesses, multiple answers possible), premises were in state that did not allow sale of products or services (164 businesses) and lack of electricity (111 businesses). The total income loss caused by these events over two years is 18 558 000.00 RWF (around $25 800.00). This amount represents tangible indirect damage costs for the SMEs. Intangible indirect damages were not touched upon in the survey but can include reputational damages, the inconvenience of experiencing the flood recovery or even mental health impacts through experiencing the disaster.

**TABLE 5:** Estimation of indirect damage costs based on the most severe event for each enterprise by year cross-analysed with estimated loss of profits during business closures after flooding (all amounts in Rwandan franc).

Indirect damage in RWF	2013	Costs 2013†	2014	Costs 2014†	Total cost†
< 1000	17	8500.00	29	14 500.00	23 000.00
1001–10 000	42	225 000.00	27	135 000.00	36 000.00
10 001–50 000	60	1 500 000.00	17	425 000.00	1 925 000.00
50 001–100 000	25	1 875 000.00	15	1 125 000.00	3 000 000.00
100 001–500 000	37	9 250 000.00	1	250 000.00	9 500 000.00
500 001–1 000 000	1	750 000.00	0	0.00	750 000.00
> 1 000 000	2	3 000 000.00	0	0.00	3 000 000.00

**Total**	**184**	**16 608 500.00**	**89**	**1 949 50.000**	**18 558 000.00**

RWF, Rwandan franc.

† , Estimated damage costs are based on the middle amount within each category (e.g. 5000 for the category 1001–10 000) and their related frequencies per year, whereas for the lowest category, an amount of 500 was used and 1.5 million for the highest category.

The total estimated direct and indirect damage costs for the businesses surveyed add up to 139 308 500.00 RWF (around $194 000.00) in 2013 and 24 049 500.00 RWF (around $33 500.00) in 2014 – based on the most severe flooding event that each affected business experienced in these two years.

To interpret these figures, it is important to take into account that the estimated damage costs are solely based on the most severe flood events experienced by the businesses surveyed. Considering the frequency of businesses being affected by floods, the real damage costs are very likely to be significantly higher than these estimates. This leads to the conclusion that in the years 2013 and 2014, floods had seriously affected the economic welfare of businesses in the surveyed area, reducing profits and causing damages in a way that was threatening the bottom line of many businesses in a serious way: For 2013, the total damage costs resemble 22% of the total annual net profit of the interviewed businesses in the area. On average, each affected business in 2013 suffered direct and indirect flood damage costs of around 737 000.00 RWF (around $1030.00) which is more than the annual net profit of around 25% of the businesses in the area.

It is remarkable that the total damage costs in 2014 were 83% lower than in 2013. There are two likely explanations for this difference: after a flood on the main road from the city centre to Nyabugogo in September 2013, local authorities took action between October 2013 and April 2014 and unblocked important waterways in the area and reconstructed a major drainage channel connected to the Nyabugogo River, spending about 265 000 000.00 RWF (around $370 000.00; Kubwimana 2014). Weather data recorded at the only weather station in Kigali (at the airport) shows that 2013 was a very wet year, while in 2014 the amount of precipitation at the weather station was only average (both compared against the overall average of 2009 to 2015); in 2014, the weather station recorded around 30% less annual rainfall than in 2013. It is likely that both the improvements in the drainage system and the low precipitation have contributed to the reduced damage costs, but it seems that the infrastructure work played a much more important role: while 2013 saw more precipitation overall, the amount of days with intense rainfall of more than 20 mm in 2013 and 2014 is the same (8 nonconsecutive days in each year; based on a data set from National Oceanic and Atmospheric Administration (NOAA)^3^ with data on precipitation in Kigali for around 30% of the days in each year). As the number of days with extreme precipitation in Kigali is similar for both years and their spread across the year is not more or less dense, one can take this as an indication that the infrastructure upgrade has made a significant contribution to reducing damage costs from flooding for businesses. In 2014, however, 45% of businesses still suffered from being flooded at least once. Furthermore, it needs to be mentioned that the weather data in both years are incomplete, that is, days are missing.

### Flood protection measures taken by businesses

The majority of businesses (71.3%) in the sample have implemented measures to reduce expected flood damages. The most common action was creating a flood barrier (160 SMEs), and moving important equipment and items in the store to higher grounds when flood water approached the premises (149 SMEs). Only 34 out of the 353 businesses (less than 10%) have an insurance cover that covers flood damages. One hundred and one SMEs did not implement any disaster reduction measures, while floods between 2013 and 2014 spared only 69 enterprises. This means that at least 32 enterprises affected by flooding did not prepare for or react to frequent flooding of their premises.

There were 101 businesses that did not undertake any measures for reducing flood damage costs for a variety of reasons: 18 of them mentioned that measures were too expensive, eight businesses said that they lacked the time to deal with these issues and only three SMEs reported that they lack information on how to protect their businesses. The main reasons for not taking action lie beyond lack of information or resources: 57 businesses (42.2%) said that they were not taking any actions because they do not expect any future flooding and 25 (18.5%) consider the impact as not being significant enough to prepare for. It should be noted that from the 57 enterprises that do not expect any future flooding, 31 businesses (54.4%) had previously been affected by a flood. When taking into account the above-mentioned evidence on the likely increase in extreme precipitation events, this resistance to taking action can be seen as problematic for the bottom line of these businesses and development in the surveyed area. There are two likely explanations for this resistance to taking action: respondents might not be aware that the likelihood and intensity of extreme weather events are projected to increase; or they might be overconfident, thinking that future floods will not affect the area where their business is based (Kind & Savelsberg 2016; Mullainathan & Thaler 2000). It is likely that both explanations play a role in explaining the observed reluctance to implement flood protection.

When asked about what the local or national government should do to reduce damage costs from flooding, 78.3% (278 businesses) responded that more information should be provided on how one can effectively protect one’s business from flood damages. Thus, there is a veritable demand for more information on how to properly deal with floods on an individual level. Among these 278 businesses, more than 50% have implemented flood protection measures. Hence it seems that there is a larger group of businesses that is very willing to take action and would like to have more information on how to make these actions more effective. On the other hand, there is the group of 77 businesses (21.7%) that did not reply that more information should be provided. Most businesses in this group have not implemented any flood protection measures and do not foresee any future flooding. This second, smaller group can be characterised as unworried about possibly increasing flood risks and uninterested in information on how to deal with floods. Again, it should be noted that a large share of this group has previously suffered damages from floods.

The most common request with respect to governmental action is the improvement of the drainage systems (mentioned by 85.1% of the surveyed 353 businesses). Making insurance products more affordable is requested by 43.1% businesses in the sample.

The topic of insurance also features highly among the measures that businesses are considering to implement in the future (mentioned by 60.6% of the businesses surveyed). The second most frequent action mentioned was relocating one’s business to a less flood-prone area (mentioned by 29.2% of the businesses surveyed). The later point is of particular interest, considering the frequent controversies about relocation of businesses in Kigali (see Barigye & Rutarindwa 2010).

## Conclusion and recommendations

With respect to the methodology, it can be concluded that the approach of asking businesses only about the damage costs of the most severe floods made it possible to arrive at a consistent data set that did not overstretch respondents’ memory and time available for responding to the survey. At the same time, this approach only allowed it to determine the absolute minimum of possible damages costs that businesses experienced from flooding in recent years. The actual annual costs will be significantly higher. However, the approach still allowed detecting relevant changes in damages’ costs between years.

The results of the survey showed that flooding is the most relevant natural hazard to businesses in the Nyabugogo area. In 2013, the annual damage costs for businesses amounted to more than 139 308 500.00 RWF (around $194 000.00, sum of direct and indirect damages) which poses a serious threat to economic development in the area as these damage costs resemble 22% of the total annual net profit of the interviewed businesses in the area. On average, each affected business in 2013 suffered direct and indirect flood damage costs of at least 737 000.00 RWF (around $1030.00), which is more than the annual net profit of around 25% of the businesses in the area. Given that extreme precipitations are likely to become more frequent and intense with the changing climate, action needs to be taken by public authorities and by businesses to ensure climate compatible growth in Nyabugogo and similar areas in Kigali.

Between 2013 and 2014, after demands from the national government, the local government took action and cleaned smaller drains and rehabilitated a large drainage channel in the area at a cost of around 265 000 000.00 RWF (around $370 000.00; Kubwimana 2014). The total annual damages costs for 2014 (based on the cost for the single most severe flood each business experienced between 2013 and 2014) were about 115 259 000.00 RWF lower than in 2013. As both years have a similar amount and spread of days with heavy rain, it is likely that larger parts of these reductions in damage costs can be attributed to the improved infrastructure. However, it should be mentioned again that the weather records available are incomplete. If one assumes that this was the only reason for the reductions, the infrastructure improvements were an investment with an excellent cost-benefit ratio from a public welfare perspective, with the reduced annual damage costs outweighing the investments costs after 3 years. But even with the seemingly cost-efficient infrastructure upgrade, the damages costs experienced in 2014 warrant much more action as 45% of the businesses surveyed were still affected by flooding. To reduce flood damages further, the drainage system in the area should be improved on a larger scale. This is the main request from businesses which is backed by experts as well (Munyaneza et al. 2013) and – as experience with the most recent infrastructure upgrade might show – could prove to be a very cost-efficient measure from a public welfare perspective.

Regarding the provision of information, there are two different demands among businesses: The majority of enterprises would like to receive information about how to protect their businesses effectively, while a small group of businesses are unwilling to take action because they do not see flooding as a relevant risk and thus also do not see a need to receive information on flood protection. To reduce future damage costs from flooding it thus seems advisable for public authorities to provide more information on effective protection measures to a larger group of businesses. Regarding the smaller group that does not see a requirement for taking action, there seems to be a need for raising awareness on the actual relevance of flooding, resulting costs and the effect of climate change on the frequency and intensity of heavy precipitation events. Possible overconfidence among business owners with respect to their enterprises’ vulnerability should also be addressed in these awareness-raising activities if one wants to increase the level of preparedness among businesses. Providing additional financial resources for flood protection, however, do not seem to be required.

Only a minority of less than 10% among surveyed businesses have an insurance that covers flooding. As around 60% are thinking about obtaining insurance and many are requesting affordable insurance products from the government, it should be investigated by public authorities, business associations, insurance providers and researchers in how far new and affordable insurance schemes could benefit businesses in the area.

Furthermore, nearly one-third of the enterprises would consider relocating their business to less flood-prone locations. This high willingness of business owners should receive notice in development planning processes; however, it needs to be taken into consideration that for a relocation to take place in an economically viable way, business owners need affordable and attractive alternative locations for their enterprises.

## Footnotes

1 There is no standardised definition of vulnerability. In this article, vulnerabilities are understood as ‘the characteristics and circumstances of a community, system or asset that make it susceptible to the damaging effects of a hazard’ (UNISDR 2007).

2 Using exchange rates from September 2015 all throughout this article.

3 This is an agency in the Department of Commerce that maps the oceans and conserves their living resources; predicts changes to the earth’s environment; provides weather reports and forecasts floods and hurricanes and other natural disasters related to weather.
